# Ferroptosis in Cancer Treatment: Another Way to Rome

**DOI:** 10.3389/fonc.2020.571127

**Published:** 2020-09-25

**Authors:** Yinan Wu, Chengcheng Yu, Meng Luo, Chen Cen, Jili Qiu, Suzhan Zhang, Kaimin Hu

**Affiliations:** ^1^Department of Breast Surgery, The Second Affiliated Hospital, Zhejiang University School of Medicine, Hangzhou, China; ^2^The Key Laboratory of Cancer Prevention and Intervention, China National Ministry of Education, Zhejiang University School of Medicine, Hangzhou, China; ^3^Department of Orthopedics, The Second Affiliated Hospital, College of Medicine, Zhejiang University School of Medicine, Hangzhou, China

**Keywords:** ferroptosis, cancer therapy, drug resistance, immunotherapy, lipid peroxiation

## Abstract

Ferroptosis is a newly described type of programmed cell death and intensively related to both maintaining homeostasis and the development of diseases, especially cancers. Inducing ferroptosis leads to mitochondrial dysfunction and toxic lipid peroxidation in cells, which plays a pivotal role in suppressing cancer growth and progression. Here, we reviewed the existing studies about the molecular mechanisms of ferroptosis involved in different antitumor treatments, such as chemotherapy, targeted therapy, radiotherapy, and immunotherapy. We focused in particular on the distinct combinatorial therapeutic effects such as the synergistic sensitization effect and the drug-resistance reversal achieved when using ferroptosis inducers with conventional cancer therapy. Finally, we discussed the challenges and opportunities in clinical applications of ferroptosis. The application of nanotechnolgy and other novel technologies may provide a new direction in ferroptosis-driven cancer therapies.

## Introduction

The term *ferroptosis* was coined in 2012 to define a unique iron-dependent form of programmed cell death. In ferroptotic cells, the mitochondria become smaller with increased membrane density, and the mitochondrial cristae usually decrease or disappear (1, 2). Ferroptosis helps maintain the death balance in normal cells and tissues (3). In cancer, some carcinogenic pathways can regulate the key modulatory factors in ferroptosis and induce ferroptosis of cancer cells (4–8).

The existing cancer therapies with unsatisfactory clinical efficacy always meet dilemmas to eradicate cancer cells due to the drug insensitivity or acquired resistance. In the past, inducing apoptosis was considered the main way to cause cancer cell death in conventional treatments. However, increasing studies have reported that inducing ferroptosis can significantly improve the efficacy of killing cancer cells, indicating that ferroptosis is another important way in treatment of cancer, where “all roads lead to Rome,” as the old saying goes. In this review, we summarized the molecular mechanisms and the roles of ferroptosis in different cancer therapies, as well as the challenges and opportunities in clinical applications of ferroptosis such as ferroptosis-driven nanotherapeutics, to draw a conclusion about the recent progress in ferroptosis-inducing cancer therapies and outline the future research and clinical application strategies.

## Molecular Mechanisms of Ferroptosis in Brief

**Figure 1:**
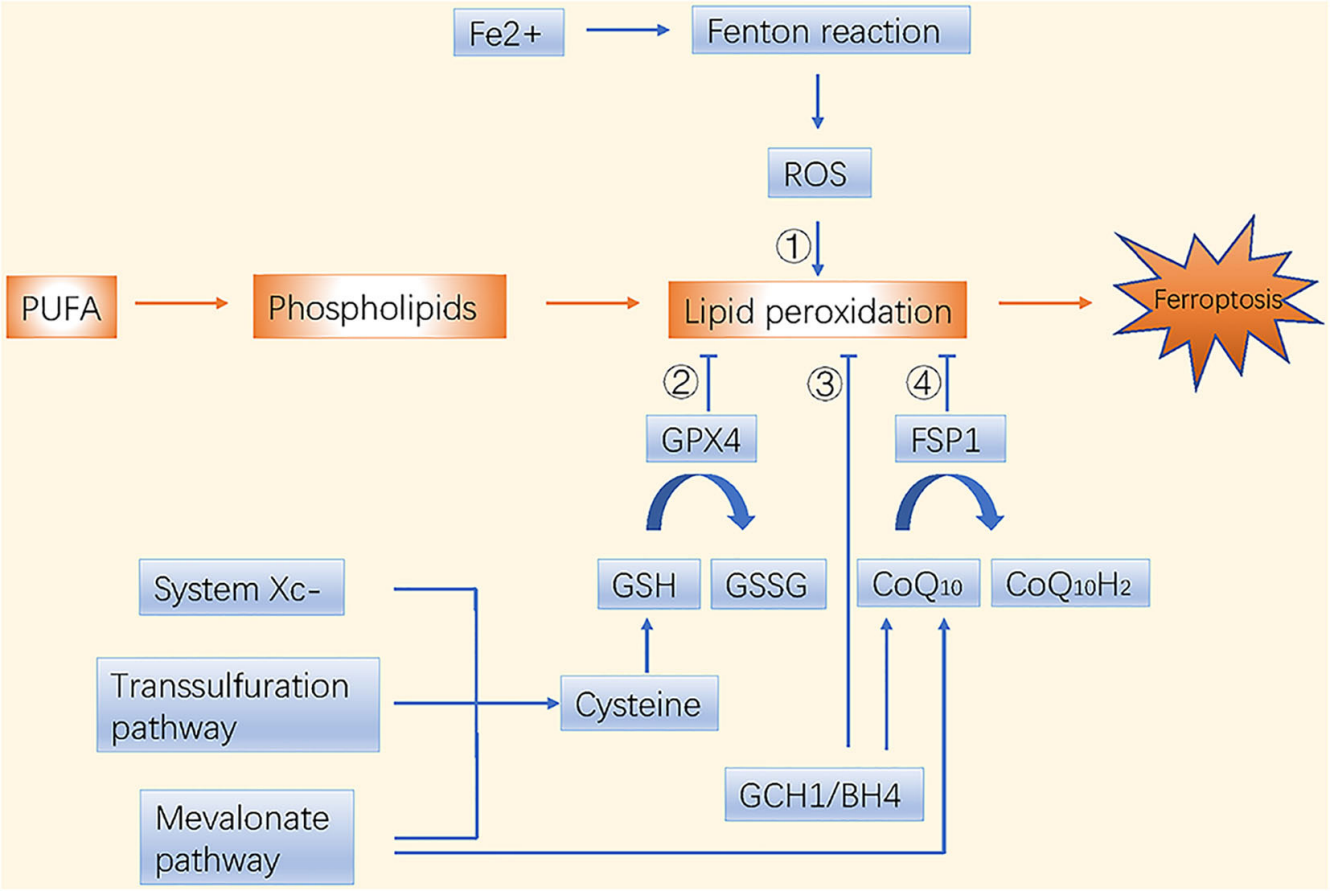
Molecular mechanisms of ferroptosis in brief. There are four main pathways to induce ferroptosis. ➀ Iron metabolism; ➁ GSH/GPX4 pathway; ➂ GCH1/BH4 pathway; ➃ FSP1/CoQ10/NAD(P)H pathway.

Dysfunctions in iron and lipid metabolism result in ferroptosis, which is characterized by accumulation of reactive oxygen species (ROS) and lipid peroxidation. Free intracellular ferrous ions react with hydrogen peroxide through Fenton reactions and activate the lipoxygenase (LOX) that induces peroxidation of polyunsaturated fatty acids (PUFAs) in cell membranes (1). The toxic free radicals generated by the oxidized lipids produce more oxidized lipids and further oxidative damage. Ferroptosis is inhibited by sequestration of free iron, inhibition of PUFA synthesis, or scavenging of ROS (Figure 1).

Ferroptosis and phospholipid peroxidation are mainly controlled by two parallel systems: glutathione (GSH)/glutathione peroxidase 4 (GPX4) and ferroptosis suppressor protein 1 (FSP1)/ubiquinone (CoQ10)/NAD(P)H axes (9, 10). The GSH/GPX4 pathway includes cystine import via the system ${\text{X}}_{\text{c}}^{-}$ (consisting of SLC7A11 and SLC3A2), cysteine production via the transsulfuration pathway, and selenocysteine production via the mevalonate pathway. Interestingly, the mevalonate metabolic pathway is crucial during the synthesis of GPX4 itself and for generation of the CoQ10 backbone. Another highly potent endogenous ferroptosis suppressor, the GTP cyclohydrolase 1 (GCH1)/tetrahydrobiopterin (BH4)/phospholipid axis, was reported recently. It inhibits ferroptosis by selectively preventing depletion of phospholipids with two PUFA tails (11). Nuclear factor erythroid 2-related factor 2 (NRF2), another endogenous antioxidant defense system, is usually maintained at a low level by tumor suppressor Kelch ECH-associated protein 1 (KEAP1)-mediated ubiquitination. Under oxidative stress, NRF2 is stabilized and activated through dissociation from KEAP1 (12); it then suppresses ferroptosis through NRF2/SLC7A11/heme oxygenase-1 (HO-1) and NRF2-FTH1 signaling (13). NRF2 is one of the central factors leading to drug insensitivity or resistance in cancer cells when coping with oxidative stress.

## The Role of Ferroptosis in Mainstream Cancer Treatments

### Ferroptosis in Classical Chemotherapy of Cancer

Ferroptosis is a new mechanism of active cancer cell death induced by classic chemotherapeutic drugs discovered in recent years (Tables 1, 2). For example, cisplatin triggers ferroptosis mainly by directly depleting intracellular GSH, which results in the consequent inhibition of GPXs (18). In addition, cisplatin can also induce ferroptosis when ferritinophagy increases free iron levels (17). In consequence, ferroptosis inducers such as erastin or RSL3 always enhance the anticancer effects of cisplatin synergistically by inhibiting system ${\text{X}}_{\text{c}}^{-}$ or GPX4 during the treatment of distinct cancer types, such as lung, colorectal, ovarian, and pancreatic ductal adenocarcinoma cancers (17, 18, 30, 31). Suberoylanilide hydroxamic acid (SAHA), a histone deacetylase inhibitor (HDACi), can also sensitize tumors to the effects of cisplatin by inducing ROS (32). Ferritinophagy-mediated ferroptosis is implicated in the therapeutic mechanisms of artemisinin (ART) and its derivatives (19–21). The iron storage protein ferritin binds to NCOA4 in the autophagosome and gets delivered into the lysosomes; ART and its derivative, dihydroartemisinin (DAT), can accumulate in the lysosomes and increase ferritin protein degradation (14), a process that increases the amount of intracellular iron and induces ferroptosis (14, 56). DAT can also regulate iron homeostasis by altering the iron-regulatory proteins (IRPs)/iron-responsive element (IRE) ratio to further increase intracellular free iron (19). Based on this, co-treatment with ART and transferrin (TF), which is responsible for the transport of iron into cells, increased lysosomal free iron and promoted ferroptosis in pancreatic ductal adenocarcinoma (PDAC) (15).

**Table 1:** Classical anti-tumor drugs and compounds associated with ferroptosis.

**Drugs/Compounds**	**Cancer types**	**Target**	**Mechanism**	**Function**	**References**
Artesunate	Cervical adenocarcinoma; Hepatocellular carcinoma	Ferritinophagy	Iron metabolism	Induce	(14)
	Pancreatic cancer	Ferritinophagy	Iron metabolism	Induce	(15)
	Head and neck cancer	NRF2	Lipid peroxidation	Inhibit	(16)
Cisplatin	Lung cancer	Ferritinophagy	Iron metabolism	Induce	(17)
	Colorectal cancer; NSCLC	GSH-GPXs; IREB2	Lipid peroxidation; Iron metabolism	Induce	(18)
Dihydroartemisinin	Lung cancer; Colorectal cancer	Ferritinophagy	Iron metabolism	Induce	(19)
	Head and neck cancer	Ferritinophagy	Iron metabolism	Induce	(20)
	Acute myeloid leukemia	Ferritinophagy	Iron metabolism	Induce	(21)
Gemcitabine	Pancreatic cancer	GPX4	Lipid peroxidation	Inhibit	(22)
Paclitaxel	Colorectal carcinoma	P53:SLC7A11	Lipid peroxidation	Unknown	(23)
	Lung cancer	P53	Lipid peroxidation	Unknown	(24)
Sulfasalazine	Sarcoma; Colorectal cancer	SLC7A11	Lipid peroxidation	Induce	(25)
	Glioma	SLC7A11	Lipid peroxidation	Induce	(26)
Sorafenib	Hepatocellular carcinoma	NRF2; SLC7A11; FTH	Lipid peroxidation; Iron metabolism	Induce	(27)
	Sarcoma	System Xc-	Lipid peroxidation	Induce	(28)
	Sarcoma; Colorectal cancer	SLC7A11	Lipid peroxidation	Induce	(25)
	Glioma	SLC7A11	Lipid peroxidation	Induce	(26)
Temozolomide	Glioblastoma multiforme	SLC7A11; Transsulfuration pathway	Lipid peroxidation	Inhibit	(29)

*NSCLC, non-small cell lung cancer*.

**Table 2:** The synergistic effect of anti-tumor therapy together with ferroptosis inducers.

**Treatment**	**Drugs**	**Drugs**	**Target**	**Cancer type**	**Mechanism**	**References**
Chemotherapy	Cisplatin	RSL3	GPX4	Lung cancer	Inhibition of GPX4 via RSL3 could enhance the anticancer effect of cisplatin	(17)
		Erastin	GSH-GPXs	NSCLC	Cisplatin can deplete the GSH and inactivate the GPXs together with erastin	(18)
				Colorectal cancer		
		Erastin	System Xc-	PDAC	Both SLC7A11-KO cell lines exhibit amino acid stress with induction of ATF4 and cell death	(30)
		Erastin	System Xc-	Ovarian cancer	Erastin can inhibit system Xc- and potentiate the cytotoxic effects of cisplatin to eradicate tumor cells	(31)
		Erastin/SAHA	ROS	NSCLC	SAHA and erastin, the inducers of ROS-mediated cell death, strongly enhanced the effect of cisplatin in WT EGFR cells	(32)
	Gemcitabine	Erastin	System Xc-	PDAC	Both SLC7A11-KO cell lines exhibit amino acid stress with induction of ATF4 and cell death	(30)
	Paclitaxel	RSL3	System Xc-	HPSCC	The combination therapy upregulates mtp53 expression, which mediated transcriptional regulation of SLC7A11	(33)
	TMZ	Erastin	System Xc-	Glioma	Erastin sensitizes glioblastoma cells to TMZ by restraining SLC7A11 and CTH function	(26)
		ALZ003	GPX4	Glioblastoma	AR suppressor ALZ003 can inhibit TMZ-resistant glioblastoma through inhibiting GPX4	(34)
	Artesunate	Trigonelline	NRF2	Head and neck cancer	NRF2 inhibitor trigonelline can induce lipid peroxide accumulation	(16)
	Cytarabine/ara-C and Doxorubicin /adriamycin	Erastin	–	Acute myeloid leukemia	JNK and p38 cooperatively participate in cell death induced by erastin in HL-60 cells	(35)
	SSZ	CN-A and PL	–	Pancreatic cancer	PL markedly depletes GSH and may inhibit GPX activity	(36)
		PL	–	Pancreatic cancer	PL markedly depletes GSH and may inhibit GPX activity	(36)
Target therapy	Lapatinib	Siramesine	Fe	Breast cancer	The combination therapy induces ferroptosis by elevating the intracellular iron level	(37)
	Lapatinib	Siramesine	Fe	Breast cancer	The combination therapy induces ferroptosis by elevating the intracellular iron level	(37)
	Lapatinib	Siramesine	Fe, Lipid peroxidation	Glioma	The combination therapy induces ferroptosis by elevating the intracellular iron level	(38)
				Lung adenocarcinoma		
	Sorafenib	X1	Lipid peroxidation	HCC	The combination therapy increases oxidative stress and mitochondrial dysfunction through activation of JNKs	(39)
	Sorafenib	Trigonelline	NRF2	HCC	NRF2 inhibitor trigonelline can induce lipid peroxide accumulation	(27)
Radiotherapy	X-ray irradiation	Erastin	GPX4	Cervical adenocarcinom	Erastin induces ferroptosis and decreases the expression levels of GSH and GPX4 protein	(40)
				Lung adenocarcinoma		
		SSZ	System Xc-	Melanoma	SSZ decreases the intratumoral level of GSH, leading to enhanced susceptibility to radiation therapy	(41)
	Laser irradiation	Gallic acid	GPX4, Lipid peroxidation	Breast cancer	Using pre-red laser irradiation could improve anticancer effects of gallic acid through decreasing GPX4 activity	(42)
				Melanoma		
	Microbeam irradiation	IKE/RSL3/sorafinib	System Xc-, GPX4	Sarcoma	System xc- or GPX4 inhibitors synergize with cytoplasmic irradiation to induce ferroptosis by enhancing cytoplasmic lipid peroxidation	(43)
				Glioblastoma		
				Lung cancer		
	Gamma knife radiosurgery	SSZ	System Xc-	Glioblastomas	SSZ treatment significantly reduced cystine uptake and GSH levels, and significantly increased the levels of ROS	(44)
	γ-radiation	Erastin	System Xc-	Breast cancer	System Xc- enhanced GSH synthesis. GSH is used to control ROS, which are therapeutic effectors of radiation therapy	(45)
	Ionizing radiation	Anti-PD-L1/Anti-CTLA4 mAb	System Xc-	Sarcoma	ATM activated by radiotherapy and IFN derived from activated CD8+ T cells synergistically inhibited the expression of SLC7A11	(46)
				Melanoma		
				Ovarian cancer		
Other therapy	Statins	Erlotinib/Gefitinib	GPX4	Lung cancer	Statins block the synthesis of GPX4	(47)
	Acetaminophen	Erastin	Iron, Lipid peroxidation	NSCLC	Acetaminophen enhances the sensitivity of erastin-induced ferroptosis by regulating the NRF2/HO-1 signaling pathway	(48)
	Bromelain	Erastin	ACSL4	Colorectal cancer	Bromelain induces ROS-induced ferroptosis via the modulation of ACSL4	(49)
	Metadherin	ML162 /ML210	SLC3A2, GPX4	Endometrial cancer	MTDH can inhibit the activities of GPX4 and SLC3A2	(50)
		Erastin	ACSL4	Breast cancer	Bromelain induces ROS-induced ferroptosis in Kras mutant Colorectal cancer cells via ACSL4	
	SCD1 inhibitors	RSL3/Erastin PEITC	Lipid peroxidation ROS	Ovarian Cancer	Stearoyl-CoA desaturase 1 inhibitors decrease an endogenous membrane antioxidant CoQ10 CN-A and PEITC synergistically trigger ROS accumulation	(51)
	CN-A			Pancreatic cancer		(52)
		PL	ROS, GSH, GPX	Pancreatic cancer	PL markedly depletes GSH and may inhibit GPX activity	(36)
		SSZ and PL	ROS	Pancreatic cancer	PL markedly depletes GSH and may inhibit GPX activity	(36)
Immunotherapy	Dichloroacetate	Albiziabioside A	GPX4	Breast cancer	AlbA-DCA can inhibit GPX4 and eliminate M2-TAMs to suppress tumor progression	(53)
		SSZ and PL		Melonoma	PL markedly depleted GSH and may inhibit GPX activity	
	Pa	Ti	–	Melanoma	Pa and Ti cooperately polarize M2-TAMs into M1-TAMs and promote the Fenton reaction with Fe ions discharged from magnetic nanoclusters	(54)
				Breast cancer		
	Oxygen-boosted PDT	Ferroptosis inducers	–	Breast cancer	PDT induces lymphocytes infiltration in the tumor site and stimulates the secretion of IFN-γ	(55)

*PDAC, Pancreatic ductal adenocarcinoma; HCC, Hepatocarcinoma; CN-A, Cotylenin A; PEITC, Phenethyl isothiocyanate; PL, Piperlongumine; PDT, Photodynamic therapy; Pa, PD-1 antibody; Ti, TGF-β inhibitor; IKE, Imidazole ketone erastin; HPSCC, Hypopharyngeal squamous carcinoma; SSZ, Sulfasalazine; TMZ, Temozolomide; AR, Androgen receptor; WT, wild-type; EGFR, Epidermal growth factor receptor; TKI, tyrosine kinase inhibitor; PFS, Progression-free survival; NSCLC, Non-small cell lung cancer; TAM, Tumor-associated macrophage; ATM, Ataxia-Telangiectasia mutated gene; CTH, cystathionine γ-lyase*.

Traditional chemotherapy drugs can also inhibit cancerous cell death and lead to ferroptosis resistance through the upregulation of GPX4 and the system X_c−_. The heat shock 70-kDa protein 5 (HSPA5)-GPX4 pathway mediated ferroptosis resistance limits the anticancer activity of gemcitabine. Specifically, gemcitabine induces the expression of HSPA5, which in turn prevents GPX4 protein degradation and subsequent lipid peroxidation in pancreatic cancer (22). Co-treatment with gemcitabine and erastin or the genetic inhibition of the HSPA5-GPX4 pathway produced additive effects eradicating pancreatic cancer cells *in vitro* and *in vivo* (30). Temozolomide (TMZ) was reported to induce SLC7A11 expression via NRF2 and ATF4 activation pathways both at mRNA and protein levels. Moreover, it can also promote cystathionine γ-lyase (CTH) activity; CTH is a key enzyme in the transsulfuration pathway that secures the cysteine supply and GSH synthesis when SLC7A11 is blocked. This may explain why gliomas with high system ${\text{X}}_{\text{c}}^{-}$ expression are more likely to be affected by the erastin-TMZ combination therapy (29, 57). The NRF2-antioxidant response element pathway can also be activated by artesunate, another chemotherapeutic drug used in head and neck cancer (HNC) cells. Inhibition of NRF2 induces lipid lipoxidation and reverses ferroptosis resistance in HNC (16).

The anticancer effects of some other classic chemotherapy drugs are enhanced when combined with ferroptosis inducers. Previous studies showed that paclitaxel (PTX), a classic chemotherapy drug used widely in multiple cancer treatments, can both upregulate the expressions of the p53 and p21 genes and downregulate the expressions of SLC7A11 and SLC1A5 in colorectal carcinoma and lung cancer cells (23, 24). Ye and his colleagues demonstrated that the combination of PTX and RSL3 rather than each agent alone induced ferroptosis in hypopharyngeal squamous carcinoma (HPSCC) cells with mutant p53 (mtp53) through the suppression of SLC7A11. mtp53 expression and, especially, the acetylation of K98 play a key role in PTX and RSL3-induced ferroptosis (33). In addition, a low dose of erastin can dramatically promote the therapeutic effects of cytarabine/ara-C and doxorubicin/adriamycin against leukemic cells in an RAS-independent manner that depends partly on ferroptosis induction. The c-Jun N-terminal kinases (JNKs)/p38-MAPK pathway was shown to play a major role in erastin-induced cell death in leukemic cells (35). The ERK-MAPK pathway is also responsible for RAS-dependent ferroptotic cell death in solid cancer cells (58). These studies suggest that the activation of the MAPK pathway may affect the anticancer efficiency of erastin in different cancers and that exploring the specificity of MAPK substrates may provide a viable option to distinguish diverse erastin-induced types of cell death.

### Ferroptosis in Targeted Cancer Therapy

Ferroptosis also plays a role in targeted therapy for cancers by interfering with target molecules involved in the development of cancer (Tables 1, 2). Sorafenib, a multi-kinase inhibitor, can directly inhibit the system ${\text{X}}_{\text{c}}^{-}$ function and can also clear the intracellular KEAP1 proteins to activate the NRF2-SLC7A11 signaling as an adaptive response (27, 59, 60). As mentioned previously, cancer cells with high expression of NRF2 can use these protective detoxification systems to prevent lipid peroxide accumulation and survive. Indeed, treatment with the NRF2 inhibitor trigonelline can promote sorafenib-induced ferroptosis in hepatocellular carcinoma cells (27). In addition, sorafenib can also block the formation of BECN1-SLC7A11 complexes and induce ferroptosis through AMP-activated protein kinase activation (25, 26, 61).

VDACs (voltage-dependent anion channels) are crucial mitochondrial membrane proteins, which can be activated to open by the erastin-like compound X1, contributing to lipid peroxidation (58). Sorafenib together with opening of VDACs increases oxidative stress and mitochondrial dysfunction through activation of JNKs in hepatocarcinoma cells (39). Moreover, sorafenib can also induce ferroptosis by triggering the expression of cathepsin B through MEK-ERK signaling phosphorylation, while inhibiting the signal transducer and activator of transcription 3 (STAT3) in human PDAC cell lines (60, 62, 63).

Siramesine combined with lapatinib can induce ferroptosis by elevating the intracellular iron level and subsequently increasing ROS in breast cancer cells (64). This specific cell death was initially induced by ferroptosis, but was regulated by spontaneous ferritin degradation and subsequent upregulation of transferrin (37). The same phenomenon has been observed in glioma and lung adenocarcinoma cell lines; the whole death process was regulated by lysosomal iron release and the degradation of HO-1by the proteasome system (38).

### Ferroptosis in Cancer Radiotherapy

**Figure 2:**
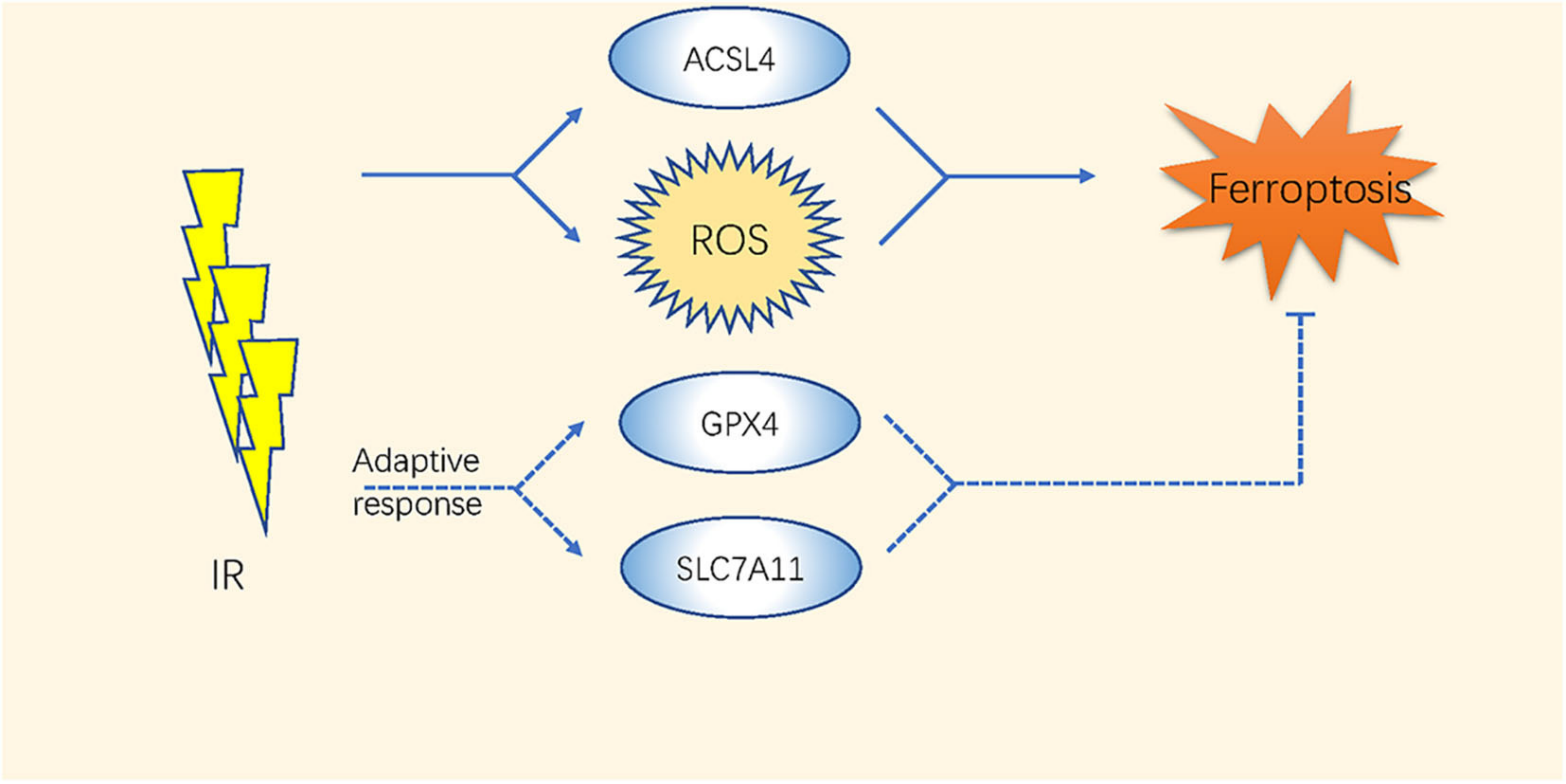
The mechanism of ferroptosis in radiotherapy of cancer.

The high-energy ionizing radiation (IR) of radiotherapy always induces direct DNA double-strand breaks (65). Moreover, indirect effects caused by the radiolysis of cellular water and the stimulation of oxidases result in GSH depletion and ROS generation (66–68) (Table 2 and Figure 2). Studies have indicated that the efficacy of radiotherapy increases when GSH is depleted (69, 70).

The expression level of SLC7A11 is higher in either glioblastoma (GBM) patient biopsies or glioma cell lines than in normal brain tissues (44); thus, inhibiting system ${\text{X}}_{\text{c}}^{-}$ or GPX4 by ferroptosis inducers enhances the therapeutic effect of radiation in breast cancer, sarcoma, and glioblastoma (40, 41, 43–45). Khorsandi et al. showed that the use of gallic acid (GA, a natural polyhydroxy phenolic compound) after pre-irradiation can significantly reduce the survival rate of breast cancer and melanoma cells mainly through inhibition of GPX4 activity without affecting normal cells (42).

Recently, Lei et al. found that IR also upregulates the expression of ferroptosis marker genes such as PTGS2 in most of the cancer cell lines. IR induces ROS directly by inducing the ACSL4/LPCAT3/ALOX and SLC7A11/GPX4 pathways as an adaptive response. Inactivating SLC7A11 or GPX4 improves the radiosensitivity of cancer cells and xenograft tumors to IR (71). Additionally, ferroptosis has been considered as a direct link to improve tumor control after co-treatments with radiotherapy and CD8+ T cells. The ataxia-telangiectasia mutated gene (ATM) activated by radiotherapy and interferon (IFN) derived from activated CD8+ T cells synergistically inhibited the expression of SLC7A11 (46). These comprehensive studies took a novel direction in improving the therapeutic effects of radiation therapy or fighting against radiotherapy resistance through the regulation of ferroptosis.

### Potential of Non-Traditional Ferroptosis Sensitizers in Cancer Treatment

Inhibiting GPX4 is a recognized way to induce ferroptosis, while mevalonate blockers are also effective. Statins, such as fluvastatin, lovastatin, and simvastatin, are small molecular inhibitors of 3-hydroxy-3-methyl-glutaryl-CoA reductase (HMGCR); they can inhibit GPX4 by regulating selenoprotein biosynthesis and CoQ10 through the mevalonate pathway, but they cannot be rescued by lipophilic antioxidants (72, 73). After treatment with fluvastatin, the expression of GPX4 decreased in a time-and concentration-dependent manner, and the effect was enhanced by combination with the direct GPX4 inhibitor RSL3 (5). Several previous clinical studies indicate that co-treatment with EGFR-TKIs and statins can improve the therapeutic effect and prolong patients' survival compared with treatment with EGFR-TKIs alone regardless of whether EGFR is mutated (47, 74, 75). It will be interesting to study the role of other mevalonate blockers in ferroptosis-mediated cancer treatments (Table 2).

Moreover, numerous promising therapeutic drugs have emerged for cancer therapy. Acetaminophen enhances the sensitivity of erastin-induced ferroptosis by regulating the NRF2/HO-1 signaling pathway (48). Bromelain induces ROS-induced ferroptosis in Kras mutant colorectal cancer cells via the modulation of ACSL4 (49). Metadherin (MTDH) can inhibit the activities of GPX4 and SLC3A2 (50). Stearoyl-CoA desaturase 1 inhibitors decrease an endogenous membrane antioxidant CoQ10 and make ovarian cancer cells prone to ferroptosis (51). Prominin2 augments the formation of ferritin-containing multivesicular bodies (MVBs) and exosomes to decrease the intracellular iron levels, thereby inhibiting ferroptosis (76). All of these agents combined with ferroptosis inducers (such as RSL3, erastin) can enhance the ferroptosis effect in different cancer cells. Cotylenin A (CN-A, a plant growth regulator) and phenethyl isothiocyanate (PEITC, a dietary anticarcinogenic compound and an inducer of ROS), have been reported to synergistically inhibit the progression of pancreatic cancer via the elevation of ROS (52). The use of CN-A alone did not remarkably affect cell viability, while the use of piperlongumine (PL) alone moderately decreased the viability to 55–70%. When used together, CN-A remarkably enhances PL-induced ferroptosis. In addition, SSZ also promotes PL-induced ferroptosis in pancreatic cancer cells. Moreover, SSZ further enhanced the cytotoxicity of mouse embryonic fibroblasts (MEFs) and cancer cells induced by PL combined with CN-A (36) (Table 2).

### Ferroptosis: a Sword to Reverse the Acquired Drug Resistance in Cancer

The mechanisms of drug resistance in cancer treatment are complex and diverse, restraint of ferroptosis is proved to be an important one.is proved to be an important one (Table 3). When treated with PTX or doxorubicin, activated NRF2 in breast and lung cancers increased drug resistance (89, 90). Improper activation of NRF2 by a mutated KEAP1 or upregulation of P62 was a crucial cytoprotective mechanism against ferroptosis (27, 91). Upregulated NRF2/FTH1 or NRF2/SLC7A11 pathways resulted in ferroptosis resistance (27, 92, 93). Cancer cells with high levels of GSH and system ${\text{X}}_{\text{c}}^{-}$ expression always show multidrug and radiation resistance (44, 92–94). Therefore, exploring the effect of small molecule inhibitors targeting the KEAP1/NRF2 pathway in cancer is of therapeutic significance. Ferroptosis agonists (erastin/sorafenib and others) and STAT3 inhibitor BP-1-102 can induce ferroptosis of cisplatin-resistant NSCLC cells via NRF2/SLC7A11 or STAT3/NRF2/GPX4 signals (31, 77, 78). ART was shown to induce ferroptosis in cisplatin-resistant HNC cells when combined with NRF2 inhibitors (16), while its derivative DAT can enhance RSL3-induced ferroptosis by increasing the intracellular free iron level in highly ferroptosis-resistant cancer cells (19). Withaferin A can induce ferroptosis by simultaneously inhibiting both GPX4 and KEAP1 proteins; the consequent upregulation of NRF2 leads to an increase in intracellular labile Fe ions upon excessive activation of HO-1 in chemo-resistant neuroblastoma cells (79).

**Table 3:** Reversing anti-tumor therapy resistance together with ferroptosis inducers.

**Treatment**	**Drugs**	**Drugs**	**Target**	**Cancer type**	**Mechanism**	**References**
Chemotherapy	Cisplatin	Erastin	ROS	NSCLC	SAHA and erastin can strongly enhance the effect of cisplatin in WT EGFR cells	(32)
		Erastin	System Xc-	Ovarian cancer	Erastin can inhibit system Xc– and potentiate the cytotoxic effects of cisplatin to eradicate tumor cells	(31)
		Erastin/sorafenib	ROS	NSCLC	Erastin/sorafenib induces cisplatin-resistant lung cancer cell ferroptosis through inhibiting the NRF2/SLC7A11 pathway	(77)
		Erastin/RSL3/STAT3 inhibitor	GPX4	Osteosarcoma	Impairing STAT3/NRF2/GPX4 signaling enhances the sensitivity of osteosarcoma cells to cisplatin	(78)
		Gefitinib/Erlotinib	ROS	Lung cancer	Inhibition of EGFR-ERK/AKT by gefitinib activates FOXO3a, which in turn reduces ROS	(32)
	Cisplatin/etoposide	Withaferin A	GPX4	Neuroblastoma	Inhibition of GPX4 by withaferin A efficiently kills chemo-resistant neuroblastoma cells	(79)
	Gemcitabine	CN-A and PEITC	–	Pancreatic cancer	The combined treatment with CN-A and PEITC synergistically increases the ROS levels	(52)
	TMZ	ALZ003	GPX4, Lipid peroxidation	Glioblastoma	AR suppressor ALZ003 can inhibit TMZ-resistant glioblastoma through inhibiting GPX4	(34)
		Erastin	GPX4, System Xc-, Transsulfuration pathway	Glioblastoma multiforme	TMZ-inducible system Xc- upregulation and CTH activation are involved in TMZ-resistance. Erastin can block SLC7A11 and reduce CTH activity	(29)
	Docetaxel	Erastin	SLC7A11	Ovarian Cancer	Erastin can conspicuously reverse the ABCB1-mediated DTXL resistance by limiting the drug efflux activity of ABCB1	(80)
		BLZ945	CD8+T cell	Ovarian Cancer	BLZ945 together with docetaxel can increase the infiltration of CD8+ T cells in tumor tissues	(81)
	SSZ	Dyclonine	ALDH, GSH	HNSCC	Simultaneous inhibition of ALDH and depletion of GSH leads to accumulation of the cytotoxic aldehyde 4-HNE and consequent cell death	
		Oxyfedrine	ALDH, GSH	Colorectal cancer		(82)
				HNSCC		
	DAT	RSL3	Ferritin	Lung cancer	DAT induces lysosomal degradation of ferritin and impinges on IRP/IRE-controlled iron homeostasis to increase cellular free iron	(19)
				Colorectal cancer		
				Breast cancer		
	Artesunate	Cisplatin	Fe	Head and neck cancer	Artesunate activates the NRF2-ARE pathway which contributes to ferroptosis resistance	(16)
Target therapy	Lapatinib	RSL3/ML210	GPX4	Breast cancer	Persistent cells acquire a dependency on GPX4. Loss of GPX4 function results in selective ferroptosis in persistent cell	(83)
	Vemurafenib	Erastin/RSL3	System xc-, GPX4	Melanoma	The combination treatment of erastin and RSL3 results in an increase in ferroptosis sensitivity	(7)
	Gefitinib/erlotinib	Cisplatin	ROS	Lung cancer	Inhibition of EGFR-ERK/AKT by gefitinib activates FOXO3a, which in turn reduces ROS	(32)
Radiotherapy	X-ray irradiation	Oxyfedrine	GSH	Colorectal cancer	Simultaneous inhibition of ALDH and depletion of GSH leads to accumulation of the cytotoxic aldehyde 4-HNE and consequent cell death	(82)
				HNSCC		
		Erastin	GPX4	NSCLC	GPX4 expression is increased in the radioresistant cells and erastin inhibits GPX4 expression	(84)
		Erastin/SSZ/RSL3/ ML162/FIN56	System xc-, GPX4, ACSL4	NSCL	IR- or KEAP1 deficiency-induced SLC7A11 expression promotes radio-resistance through inhibiting ferroptosis	(71)
				Esophageal cancer		
	RT-MPs	Anti-PD-1	–	Lung carcinoma	RT-MPs polarize M2-TAMs into M1-TAMs. TAMs increase the expression of PD-L1, which enhances the follow-up effect of combined therapy with anti-PD-1	(85)
				Melonoma		
Other therapy	Lovastatin	Gefitinib	GPX4	NSCLC	Down-regulation of RAS protein leads to inhibition of both the RAF/ERK and AKT pathways	(86)
	Atorvastatin	Gefitinib	GPX4	NSCLC	Down-regulation of RAS protein leads to inhibition of both the RAF/ERK and AKT pathways	(87)
	Simvastatin	Erlotinib/Gefitinib	GPX4	NSCLC	Statin may overcome the EGFR-TKI-resistance through AKT/β-catenin signal-dependent reduction of surviving	(88)
	Prominin2	RSL3/ML210/ FIN56/Erastin	Ferritin	Breast cancer	Prominin2 promotes the formation of ferritin-containing MVBs and exosomes that transport iron out of the cell	(76)

*RT-MPs, Irradiated tumor cell-released microparticles; SSZ, Sulfasalazine; TMZ, Temozolomide; CN-A, Cotylenin A; PEITC, Phenethyl isothiocyanate; HNSCC, Head and neck squamous cell carcinoma; NSCLC, Non-small cell lung cancer; EGFR, Epidermal growth factor receptor; TKI, tyrosine kinase inhibitor; DAT, Dihydro-artemisinin; AR, Androgen receptor; WT, wild-type; TAM, Tumor-associated macrophage; MVBs, multivesicular bodies*.

Drug-resistant cells also tend to express more proteins involved in iron metabolism, such as transferrin receptor (TFR) and ferritin and less ferroportin-1 (FPN, the iron transport protein) than drug-sensitive cells (95–98). In general, increased iron uptake (e.g., due to TFR overexpression), reduced iron storage (e.g., due to the knockdown of ferritin or induction of ferritinophagy), and impaired cellular iron export (e.g., resulting from the knockdown of FPN) enhance the sensitivity to ferroptosis (99). On the other hand, a decrease in intracellular free iron leads to ferroptosis resistance. Targeting TFR, ferritin, or FPN strategies with anticancer therapies are highly effective for overcoming the chemo-resistance of cancer cells. An article published in 1993 showed that downregulation of TFR reversed drug resistance in cancer cells (100). Wu et al. later reported that combination therapy with anticancer drugs and TFR targeting is highly effective for overcoming the chemo-resistance of hematologic malignancy cells (101). Bortezomib prevents the upregulation of ferritin in response to iron, thus limiting the ability to buffer ROS. Reduction of basal ferritin levels or iron supplementation increases cell death and overcomes bortezomib resistance (102). Targeting the downregulation of ferritin L subunit by miR-133a increases the sensitivity of chemo-resistant breast cancer cells to cisplatin and doxorubicin (97). These results suggest that regulating the iron metabolism balance may substantially improve the efficacy of cancer treatments. Elevated levels of aldehyde dehydrogenases (ALDHs) in cancers are a mark of treatment resistance and an effective target for ferroptosis induction. Sulfasalazine (SSZ)-resistant head and neck squamous cell carcinoma (HNSCC) cells often present high expression of ALDH3A1, and genetic or pharmacological inhibition of ALDH3A1 can make these cells regain their sensitivity to SSZ (103). Dyclonine (DYC) and oxyfedrine (OXY), the covalent inhibitors of ALDHs, can cooperate with GSH-depleting agents such as buthionine sulfoximine (BSO) or SSZ to induce intracellular accumulation of the toxic aldehyde 4-hydroxynonenal (4-HNE), a specific lipid peroxidation by-product, and suppress the growth of SSZ-resistant gastric tumors or HNSCCs with high ALDH3A1expression (82, 103).

A recent study showed that drug-resistant cancer cells in a high-mesenchymal cell state were sensitive to ferroptosis induced by GPX4 suppression or statin treatment (5). Similarly, targeting of HER2-amplified breast cancer cell lines with lapatinib leaves a population of persistent cells acting as high-mesenchymal state cells. A remarkable feature of this group of cells is that they depend on GPX4 for survival; thus, GPX4 inhibitors further eradicated this population of residual cells (83). Persistent melanoma cells induced by the BRAF kinase inhibitor vemurafenib also resulted in an increase in ferroptosis sensitivity upon both erastin and RSL3 treatments (7). FSP1, a key component of a non-mitochondrial coenzyme Q10 (CoQ10) antioxidant system, acts in parallel to the GSH/GPX4 pathway. It reduces CoQ10, which acts as a lipophilic radical-trapping antioxidant that halts the propagation of lipid peroxides and inhibits ferroptosis (9). Furthermore, Doll et al. found tumor cells with GPX4_KO_ and FSP1 overexpression that were insensitive to ferroptosis-inducing agents. Treatment with a type of FSP inhibitor (iFSP) selectively induced ferroptosis in these cells (10).

Ferroptosis can also reverse chemo-resistance through other mechanisms. A combined therapy of CN-A and PEITC can trigger ROS accumulation and inhibit the proliferation of gemcitabine-resistant pancreatic cancer cells (52). One of the characteristics of TMZ-resistant cancer cells is the overexpression of androgen receptor (AR); co-treatment with erastin or AR suppressor significantly increased the TMZ-induced cytotoxicity and overcame TMZ resistance through GPX4 inhibition (29, 34). Similarly, overexpression of ABCB1 (P-glycoprotein/MDR1) in ovarian cancers usually leads to chemo-resistance via intracellular drug export (104, 105). Erastin can conspicuously reverse the ABCB1-mediated DTXL resistance by limiting the drug efflux activity of ABCB1 (80).

Inhibiting SLC7A11 or GPX4 sensitizes radio-insensitive cancer cells to IR (71). GPX4 expression is increased in radioresistant NSCLC cells, and knocking down GPX4 or using ferroptosis inducers that target GPX4 can enhance the sensitivity of radiation-resistant lung cancer cells after high-dose hypofractionated irradiation. Erastin and IR exhibited a combined cell killing effect compared with either erastin or IR alone (84). Moreover, co-treatment with oxyfedrine (OXY) can trigger the intracellular accumulation of lipid peroxidation and enhance cell death (82). In conclusion, triggering ferroptosis is a promising method to eradicate treatment-resistant cancer cells.

## Ferroptosis in Tumor Microenvironments and Immunotherapy of Cancer

### Immune Activation and Immunosuppression

**Figure 3:**
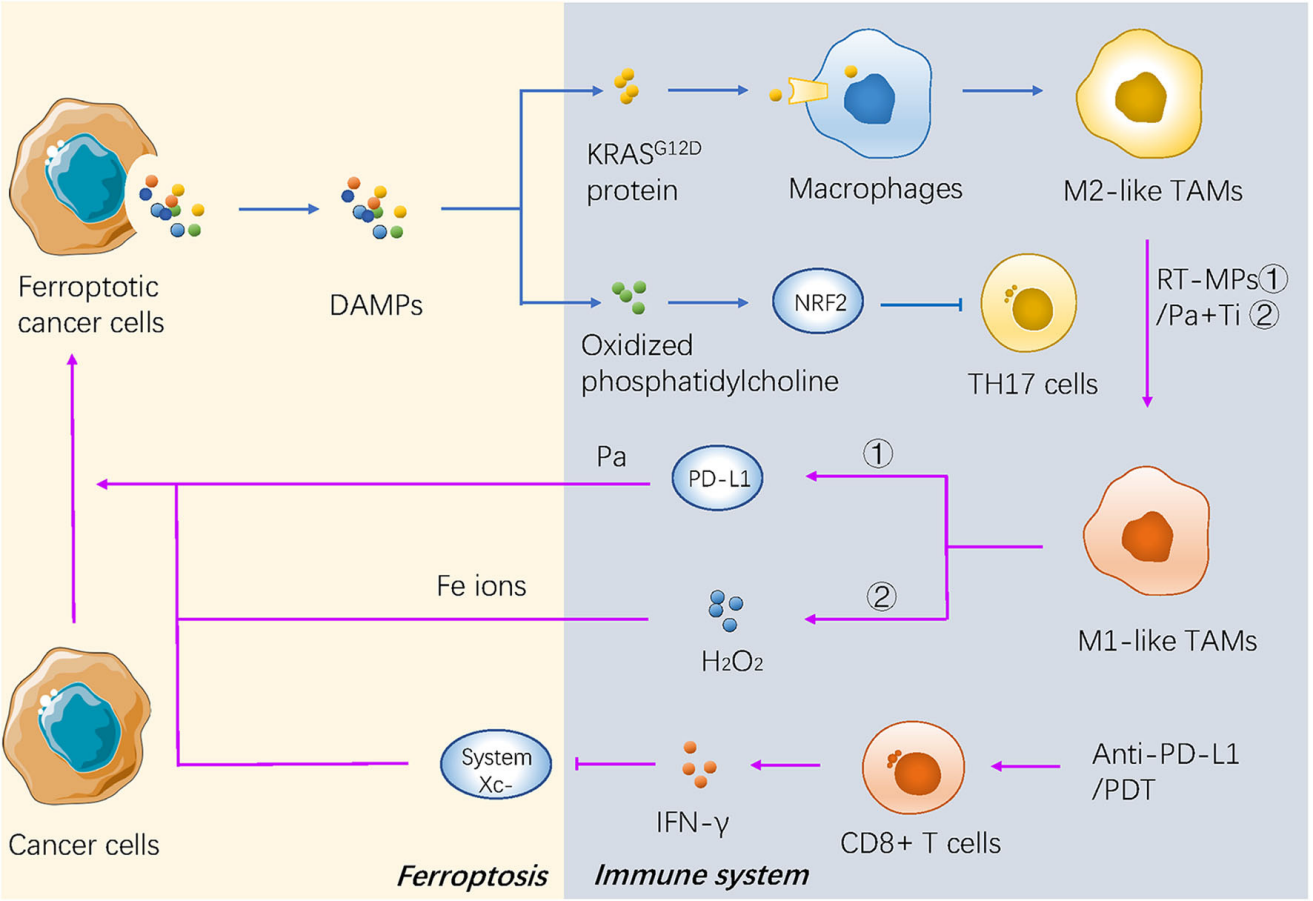
The mechanism of ferroptosis in tumor microenvironment and immunotherapy of cancer. DAMPs secreted by dying cancer cells promotes recruitment of tumor-infiltrating dendritic cells, contributing to immunogenic phagocytosis. And some kind of novel immunotherapies promote the cancerous ferroptosis in turn.

Ferroptotic cancer cells can continuously release immunostimulatory damage-associated molecular pattern molecules (DAMPs) and activate the adaptive immune system within the tumor microenvironment to enhance anticancer effects (106, 107). The phagocytosis of bone-marrow-derived dendritic cells (BMDCs) is activated by three key DAMPs (CRT, HMGB1, and ATP) from ferroptotic cells (108). Neutrophils are also recruited through a TLR4/TRIF/type 1 IFN or a Wnt signaling pathway induced by ferroptotic cancer cells (109, 110), which are important modulators and potential therapeutic targets against cancer progression (Figure 3).

Two major subtypes of tumor-associated macrophages (TAMs) are generated depending on different environmental stresses, including antitumor M1 and procarcinogenic M2 TAMs (111). The KRAS^G12D^ protein released from ferroptotic pancreatic cancer cells is further absorbed by macrophages through an advanced glycosylation end product-specific receptor, which then induces STAT3-dependent fatty acid oxidation and drives macrophages to form M2-like TAMs (112). Oxidized phosphatidylcholine from ferroptotic cancer cells also inhibits DC maturation and inhibits the differentiation of T helper 17 (TH17) cells through activation of NRF2 (113). In addition, the pharmacological induction of ferroptosis *in vivo* may also affect the tumor microenvironment (TME). For example, the activity of GPX4 is positively correlated with the survival of immune cells. GPX4-knockout in the myeloid lineage stimulated the appearance of intestinal tumors in intestinal epithelial cells (114). Antigen-specific CD4+ and CD8+T cells lacking GPX4 are likely to die by ferroptosis (115) (Figure 3).

Taken together, the evidence suggests that the interaction between ferroptosis and the immune system is complicated, and does not just lead to a completely positive or negative effect. Therefore, understanding the mechanisms of the adaptive immune system during ferroptosis in cancer cells is difficult, but necessary to improve the efficacy of clinical anticancer therapy.

### Clinical Application of Ferroptosis in Immunotherapy

A recent study reported that IFN-γ secreted from cytotoxic CD8+ T cells downregulated the expression of system ${\text{X}}_{\text{c}}^{-}$, which makes cancer cells sensitive to ferroptosis (116), indicating that the combination therapy of ferroptosis inducers and immune checkpoint inhibitors could be a promising direction in the future. Some novel treatments also have focused on the interaction between ferroptosis and immunotherapy. Both the irradiated tumor cell-released microparticles and the combined therapy of PD-1 antibody with TGF-β inhibitor can turn M2-polarized TAMs into M1-TAMs to regulate the antitumor interactions between tumor cells and TAMs (85). Nanoparticles with an IO nanocluster core encapsulated in engineered leukocyte membranes containing PD-1 antibody and TGF-β inhibitor were designed; PD-1 antibody and TGF-β inhibitor cooperatively enhanced the immune response in the TME, resulting in an increase in the level of H_2_O_2_ in M1 polarized macrophages and triggering Fenton reactions with Fe ions released from the core of the nanoparticles. The generated hydroxyl radicals subsequently induce lethal ferroptosis to tumor cells (54). A novel conjugate AlbA-DCA was developed via inhibition of the GPX4 pathway and elimination of procarcinogenic M2-TAMs to suppress tumor progression in breast cancer (53) (Table 2 and Figure 3). Another study reported that M1 phagocytes exert higher ferroptosis resistance than M2 phagocytes, but that inducible nitric oxide synthase (iNOS)/NO-enrichment of phagocytes can modulate the sensibility of phagocytes to ferroptosis. iNOS depletion/inactivation makes M1 cells sensitive to ferroptosis, while NO donors promote ferroptosis resistance of M2 cells (117). Combining immunotherapy with irradiation can even reverse the chemo-resistance of cancer cells. As previously mentioned, RT-MPs can polarize M2-TAMs into M1-TAMs, which have been reported to release H_2_O_2_ (118). When taken up by tumor cells, RT-MPs can cause immunogenic cell death mainly through ferroptosis, thereby making cisplatin-resistant tumor cells more vulnerable to attack by macrophages (85).

Photodynamic therapy is based on photosens (PS-PDT), which are potent inducers of both ferroptosis and phenotypic maturation of BMDCs in two different cancer cell lines (108). After the combination therapy of oxygen-boosted PDT and potent ferroptosis inducers (a 2-in-1 nanoplatform constructed with hemoglobin, photosensitizer chlorin e6, and sorafenib), the level of GSH was decreased while MDA, one of the last-step products of lipid peroxidation, was increased in tumor tissues (corroborating the effects of ferroptosis). In addition, PDT upregulated the level of lymphocyte infiltration in the tumor site and stimulated secretion of IFN-γ (55). As mentioned above, IFN-γ secreted from cytotoxic CD8+ T cells can downregulate the expression of system ${\text{X}}_{\text{c}}^{-}$ and induce ferroptosis in cancer cells (116); thus, PDT stimulation could result in both ferroptosis and regulation of the immune TME (Table 2 and Figure 3).

## Ferroptosis-Driven Nanotherapeutics and Other Novel Ways

Although researches on ferroptosis in cancer therapy have progressed rapidly in recent years, great problems for its clinical application remain, such as the low solubility and poor membrane permeability of ferroptosis-inducing anticancer drugs or the off-target toxicity to normal cells and tissues (119–121). In view of this plight, development of ferroptosis-driven nanotherapeutics seems to be a promising stratage (122). Ferroptosis-driven nanotherapeutics work in three main ways: First, they can trigger or promote Fenton reactions in tumor cells (123). Nanoparticles are always designed to provide two basic elements of the Fenton response: Fe ions and (or) H_2_O_2_. Ferroptosis-combined cancer therapy is mainly based on iron-containing nanomaterials. The hypoxia-induced lower pH inherent to TMEs allows easy release of Fe ions from iron-containing nanomaterials, which triggers the Fenton reaction and leads to ferroptosis in cancer cells. Second, ferroptosis-driven nanotherapeutics are used as carriers of chemotherapeutic drugs or ferroptosis inducers to inhibit the expression of the GSH/GPX4 system in tumor cells (124). For example, sorafenib can be encapsulated in a nanostructure consisting of Fe ions. The suppression of GPX4 by sorafenib combined with the tumor site-specific Fenton reaction induced by Fe ions results in a powerful tumor-specific ferroptosis (125). Nano-carriers can also enhance the internalization of platinum and iron in cancer cells that can bypass the biological barrier of cisplatin (one of the main causes of cisplatin resistance), thereby overcoming this drug resistance by tumor cells (126). Third, nanotherapeutics can provide exogenous regulation of lipid peroxidation to tumor cells, including through oral PUFA supplementation or by delivering PUFAs in nanoparticulate drug delivery systems to regulate lipid peroxidation and directly induce ferroptosis in tumor cells (127, 128).

In addition to nanotherapeutics, Gao and colleagues reported another new method to overcome multidrug resistance in the treatment of cancer through tailored ferroptotic micelles, which consist of ferroptosis inducers with arachidonic acid-conjugated amphiphilic copolymers, opening a new horizon for treating drug-resistant tumors (129). Exosomes have become popular as drug carriers with low immunogenicity, high efficiency, and high biocompatibility. Folate (FA)-vectorized exosomes loaded with erastin (erastin@FA-exo) were developed recently to act on triple-negative breast cancer (TNBC) cells with high expression of FA receptors, and which can overcome the poor water solubility and nephrotoxicity of erastin and result in a better therapeutic effect (130). Moreover, a group of studies are focusing on the relationship between ferroptosis and autophagy (131), the specific association between ferroptosis and other kinds of cell death requires more in-depth research in the future.

## The Challenges in Clinical Applications of Ferroptosis

Ferroptosis is a novel potential target in cancer treatment, however, precise mechanisms in regulation of ferroptosis in cancers are sometimes complicated and still need to be further explored. For instance, we have mentioned that p53 plays an important role in PTX-induced ferroptosis through downregulation of SLC7A11 and SLC1A5. However, the role of p53 in ferroptosis remains obscure. According to the p53 mutation status and cellular context, p53 can have either pro- or anti-ferroptotic functions during oxidative stress states (132). To be specific, p53 seems to act like a rheostat, suppressing ferroptosis under low oxidative stress, and enhancing it under high ROS stress (133). Similarly, HO-1 also plays contradictory roles promoting or inhibiting ferroptosis depending on the cellular redox status. Downregulated HO-1 in mice renal proximal tubule cells induced ferroptosis, while upregulated HO-1 promoted ferroptosis induced by Bay through NRF2/SLC7A11/HO-1 signaling in breast cancer cells (134). A previous study reported that AMPK-mediated BECN1 phosphorylation promotes ferroptosis by inhibiting SLC7A11-mediated cystine transport (25). However, a recent study proposed the contradictory conclusion that cancer cells with high basal AMPK activation are resistant to ferroptosis and AMPK inactivation sensitizes these cells to ferroptosis. Functional and lipidomic analyses have further revealed that ferroptosis is regulated via AMPK-mediated phosphorylation of acetyl-CoA carboxylase and restrained biosynthesis of PUFAs or other fatty acids (135). It is possible that the function of AMPK in regulating ferroptosis is context dependent, which awaits further exploration in future studies.

In addition to the unclear mechanisms mentioned above, recent studies have also proposed new directions in this field. Previous research indicated that the system ${\text{X}}_{\text{c}}^{-}$/GSH/GPX4 axis was the key pathway in ferroptosis, therefore, the synthesis of GSH is essential. However, inhibition of GSH synthesis did not induce lipid ROS or decrease cell viability in PDAC. Badgley et al. found that cysteine rather than GSH is a central factor in this pathway to induce ferroptosis and that PDAC cells use cysteine to synthesize both coenzyme A and GSH, which cooperatively downregulate cancerous ferroptosis (136). It was once believed that enzymatic lipid peroxidation is primarily catalyzed by arachidonate lipoxygenases (ALOXs), however, the necessity for ALOX involvement in ferroptosis induction was questioned by a recent identification of previously unknown radical-trapping activities of ALOX-inhibiting small molecules used to establish the ALOX-ferroptosis connection (137), but Zou et al. firstly confirmed that cytochrome P450 oxidoreductase (POR), a distinct ferroptosis inducer, is necessary for ferroptosis through peroxidation of polyunsaturated phospholipids on the cell membrane. The pro-ferroptosis effect of POR is applicable to various types of cancer lineages (138).

The complexity of biological system and the difficulty in clinical translation bring challenges and opportunities to the further development of ferroptosis-based cancer therapies concomitantly. The following concerns should be taken into account when applying treatments targeting ferroptosis: (1) Successful experiments *in vitro* or animal models cannot be directly applied to clinical practice. Clinical trials are needed to be conducted to evaluate the safety and practical efficacy of ferroptosis-inducing therapies. (2) Ferroptosis as a double-edged sword can not only be used as an effective therapeutic target, but also may cause cancers and other diseases. Therefore, special attentions should be paid about the dose, duration and tissue specificity of ferroptosis-inducing drugs to destroy cancer cells and avoid off-target toxicity to normal cells and tissues. (3) Some intrinsic characteristics of available ferroptosis inducers, such as the limited water solubility and toxic effects on kidneys, eyes and the immune systems restricted their clinical applications (119–121). As previously mentioned, antigen-specific CD4+ and CD8+T cells lacking GPX4 are likely to die by ferroptosis (115), and GPX4-knockout in the myeloid lineage can even induce intestinal tumors in intestinal epithelial cells (114), indicating an important role of GPX4 in maintaining a healthy immune system. Once the off-target toxicity to normal cells and tissues occurs, it brings serious aftereffects. Deferoxamine is a classic ferroptosis inhibitor which can be used as a neutralizer to avoid excessive ferroptosis and injury to normal cells and tissues. However, the short half-life of which limits its clinical application, indicating an urgent need to develop an effective and biocompatible novel ferroptosis inhibitor (139). Nanoscale material is another good choice to solve the problem about low efficiency and high side effects of traditional ferroptosis inducers as well as the potential off-target toxicity to normal cells and tissues. The low toxic cisplatin prodrugs can be loaded in iron oxide nanoparticles, which are accumulated at specific site of tumors in the presence of magnetic field, and the prodrugs can be reduced to the toxic cisplatin to induce ferroptosis in cancer cells specifically (126). CY-1-4 (an anti-malaria drug tryptanthrin derivative) carried by nanoparticles can significantly improve the drug's solubility and improve its transportation efficiency *in vivo* to induce cancerous ferroptosis with a higher efficiency (140). Potential risks also existed in the ferroptosis-driven nanotherapeutics. Nanomaterials themselves have cytotoxic effects, which may lead to a greater risk of side effects in disease treatments. The possibility of cellular protective autophagy induced by some nanomaterials is another great challenge to their further clinical application. Therefore, numerous issues should be taken into consideration in the rational design of efficient and safe ferroptosis-driven nanotherapeutics in the future.

## Conclusions and Perspectives

Ferroptosis is an undeniably important manner of cell death in addition to apoptosis in cancer therapies. Here we collated and reviewed how ferroptosis was involved in the cancer treatments according to studies to date. Different therapeutics, including chemicals, targeted agents, radiotherapy and also immunotherapy, induce ferroptosis to kill cancer cells through distinct molecular mechanisms. Insensitivity and acquired resistance are the main obstacles in cancer eradication. Ferroptosis inducers are promising in the field of oncotherapy for their sensitization and synergistic effects. Integrating ferroptosis with burgeoning biotechnologies such as ferroptosis-driven nanotherapeutics may overcome the limits of traditional medicals and ferroptosis inducers with low solubility and poor membrane permeability. Due to the complexity of biological system and the difficulty in clinical translation, further studies and clinical trials are still warranted.

## Author Contributions

YW, CY, and JQ reviewed the literature and drafted the article. ML, CC, SZ, and KH finalized the paper and provided suggestions to improve it. All authors participated in designing the concept of this manuscript.

## Conflict of Interest

The authors declare that the research was conducted in the absence of any commercial or financial relationships that could be construed as a potential conflict of interest.
